# Ecological selection of siderophore‐producing microbial taxa in response to heavy metal contamination

**DOI:** 10.1111/ele.12878

**Published:** 2017-11-21

**Authors:** Elze Hesse, Siobhán O'Brien, Nicolas Tromas, Florian Bayer, Adela M. Luján, Eleanor M. van Veen, Dave J. Hodgson, Angus Buckling

**Affiliations:** ^1^ ESI & CEC, Biosciences University of Exeter Penryn Campus Cornwall TR10 9FE UK; ^2^ Institut für Integrative Biologie ETH Zürich Universitätstrasse 16 Zürich 8092 Switzerland; ^3^ Département de Sciences Biologiques Université de Montréal 90 Vincent‐d'Indy Montréal QC H2V 2S9 Canada; ^4^ CIQUIBIC Departamento de Química Biológica Facultad de Ciencias Químicas CONICET Universidad Nacional de Córdoba Córdoba X5000HUA Argentina; ^5^ Camborne School of Mines CEMPS University of Exeter Penryn Campus Cornwall TR10 9FE UK; ^6^ CEC University of Exeter Penryn Campus Cornwall TR10 9FE UK

**Keywords:** Adaptation, detoxification, ecological species sorting, evolution, metal tolerance, public good dynamics, remediation, selection

## Abstract

Some microbial public goods can provide both individual and community‐wide benefits, and are open to exploitation by non‐producing species. One such example is the production of metal‐detoxifying siderophores. Here, we investigate whether conflicting selection pressures on siderophore production by heavy metals – a detoxifying effect of siderophores, and exploitation of this detoxifying effect – result in a net increase or decrease. We show that the proportion of siderophore‐producing taxa increases along a natural heavy metal gradient. A causal link between metal contamination and siderophore production was subsequently demonstrated in a microcosm experiment in compost, in which we observed changes in community composition towards taxa that produce relatively more siderophores following copper contamination. We confirmed the selective benefit of siderophores by showing that taxa producing large amounts of siderophore suffered less growth inhibition in toxic copper. Our results suggest that ecological selection will favour siderophore‐mediated decontamination, with important consequences for potential remediation strategies.

## Introduction

It is becoming increasingly apparent that many public goods benefit not only conspecifics but also other species. For example, many bacterial proteases show extracellular activity, providing potential nutritional benefits to neighbouring bacteria independent of their taxonomy (Suleman 2016); and immune‐repressing molecules produced by parasitic nematodes provide a potential benefit to all co‐infecting parasites (Maizels *et al*. 2001). Regardless of whether public goods are solely conspecific or also have interspecific benefits, there is potential for non‐producers to outcompete producers assuming public good production carries some metabolic cost (Hamilton 1964; Hamilton & Axelrod 1981; Frank 1994). Hence, the evolution of costly public goods is crucially dependent on the extent to which benefits are reaped by producers, other individuals carrying the public good gene or non‐producers. While the evolution of public goods has been studied extensively within species, we know very little about how ecological species sorting influences interspecific public good production within natural communities. Here, we combine surveys and experiments to determine how ecological selection acts on a microbial interspecific public good: siderophore‐mediated heavy metal detoxification.

Heavy metals are ubiquitous components of the Earth's crust, and large amounts have been released into the environment as a result of human activities (Nriagu & Pacyna 1988). Heavy metals are toxic to microbes to varying degrees (Giller *et al*. 1998) and their presence can greatly impact natural microbial communities (Gans *et al*. 2005). In the face of long‐term selection imposed by heavy metals, microbes have evolved mechanisms to cope with metal toxicity, including metal reduction, reduced cell permeability, and extracellular sequestration (Nies 1999; Bruins *et al*. 2000; Valls & De Lorenzo 2002). One such detoxification mechanism is the production of siderophores. While the canonical function of siderophores is to scavenge insoluble iron (Ratledge & Dover 2000), bacteria also use these secreted molecules to bind other metals (Braud *et al*. 2010). Siderophore production can be induced by the presence of non‐iron metals (Hofte *et al*. 1993; Teitzel *et al*. 2006), which they bind with various affinities (Braud *et al*. 2009). These siderophore‐metal complexes are unable to enter bacterial cells, thereby reducing free toxic metal concentrations in the environment (Schalk *et al*. 2011). This has led to the suggestion of adding siderophores or siderophore‐producing microbes to remediate metal‐contaminated environments (Rajkumar *et al*. 2010; O'Brien & Buckling 2015). However, to understand how siderophores may both contribute to natural decontamination and long‐term remediation efficacy, it is crucial to determine how metal toxicity affects selection for siderophore production in natural communities.

Given their detoxifying effect, increasing metal toxicity might be expected to result in ecological sorting in favour of species with greater siderophore production. However, the production of detoxifying siderophores not only benefits the producer (or its close relatives), but potentially also neighbouring cells, both con‐ and hetero‐specific, in the community. Siderophore production – which is up‐regulated in response to heavy metals (Hofte *et al*. 1993; Teitzel *et al*. 2006) – is often associated with a fitness cost, hence selection may favour cells that produce fewer siderophores, but still receive the same detoxifying benefits of siderophore production from neighbours (West *et al*. 2007; O'Brien *et al*. 2014). This can result in a ‘tragedy of the commons’, whereby mean siderophore production levels are actually reduced in the presence of toxic metals, despite the benefits that siderophores would provide to the group as a whole (O'Brien *et al*. 2014). Moreover, the (almost) complete loss of public goods production, and the resultant decline in group productivity, has been observed in various experimental set ups, including siderophore production under iron‐limited conditions (Griffin *et al*. 2004). Limited diffusion of public goods (Kummerli *et al*. 2009, 2014) and positive assortment of producing cells resulting from spatial structure (Hamilton 1964; West *et al*. 2007; Mitri & Foster 2013; Ghoul & Mitri 2016; Pande *et al*. 2016) may, however, limit community‐wide benefits of producing and prevent overexploitation by non‐producing cells (Oliveira *et al*. 2014), potentially resulting in stable coexistence of producing and non‐producing taxa (Cordero *et al*. 2012; Morris *et al*. 2012; Morris 2015; Estrela *et al*. 2016). The situation is further complicated by the iron‐scavenging function of siderophores, which is also open to exploitation within (Griffin *et al*. 2004; Buckling *et al*. 2007; Lujan *et al*. 2015) and between species (Barber & Elde 2015; Galet *et al*. 2015). Given that siderophores provide direct benefits (and indirect benefits through helping kin), but may also benefit non‐kin and other species, it is unclear if net siderophore production will increase or decrease in natural communities as a function of metal toxicity.

To investigate how metal contamination affects ecological selection for siderophore production, we first confirmed that siderophores can act as interspecific public goods in an *in vitro* siderophore‐addition experiment. We then conducted a survey along a natural contamination gradient. We correlated total metal content and soil acidity with species composition and estimates of siderophore production determined from the proportion of bacteria that show detectable extracellular iron‐chelation *in vitro*. Soil acidity is an important environmental factor determining metal solubility and thereby toxicity. We then conducted an experimental study in compost communities to determine causal links between metal contamination and siderophore production. Note that we do not simultaneously address within species selection alongside ecological selection, largely because the genetic resolution of our sequencing methods is only at the genus level.

## Methods

### Siderophores as interspecific public goods

To test whether siderophores can act as interspecific public goods we quantified whether the presence of heterospecific siderophores – produced by taxonomically diverse soil‐dwelling microbes – ameliorates growth of non‐producing *Pseudomonas aeruginosa* in toxic copper. We inoculated ~ 10^4^ colony forming units (CFUs) of a producing *P. aeruginosa* strain (PA01) and an isogenic non‐producing mutant (PA01*ΔpvdDΔpchEF*) in isolation into 3–4 replicate micro‐centrifuge tubes, containing 900 μL of copper‐contaminated KB broth (final morality 0.6 mm CuSO_4_), which reduces relative non‐producer fitness (O'Brien *et al*. 2014). In addition, ~ 10^4^ CFUs of either strain were inoculated in copper broth containing 0.6 mm of yersiniabactin (produced by *Pseudomonas stutzeri*), ornibactin (*Burkholderia vietnamiensis*), ferrioxamine E (*Streptomyces olivaceus*) or schizokinen (*Bacillus megaterium*) (http://bertrandsamuel.free.fr/siderophore_base/siderophores.php). Copper is a common heavy metal (Nriagu & Pacyna 1988), including at our field site (Fig. 2a); hence, we used CuSO_4_ in all *in vitro* assays. Bacterial cultures were horizontally shaken at 37 °C for 24 h, after which culture was plated onto agar to obtain cell densities and calculate Malthusian growth rate: *m *= ln(N_f_/N_0_)/Δt, where N_0_ and N_f_ are initial and final bacterial densities, and Δt = 24 h.

To confirm that non‐producer growth was lower in toxic copper compared to that of the siderophore‐producing strain, we used a one‐way anova. We next tested whether heterospecific siderophores can ameliorate non‐producer growth using a one‐tailed t‐test to compare mean growth differences between strains in control and siderophore‐supplemented copper broth.

### Natural microbial communities

#### Soil collection and characterisation

Soil samples were collected in a former poly‐metallic mining area situated in the Poldice Valley (N: 50°14.56; W: 5°10.10) in Cornwall (UK). The valley is rich in heavy metals, as apparent from the significant production of heavy metals during the 18–19th centuries (Burt 1998). The area is no longer worked leaving a legacy of untreated mining waste. 94 samples were collected by pushing sterile bulb planters into the ground near chimneys, slag heaps and regenerated areas, representing a wide contamination range. The upper part of the soil core was discarded to rule out possible ground surface contamination. Samples were then transferred to sterile 50 mL falcon tubes and stored at 4 °C until further processing. Prior to DNA extraction and soil characterisation, samples were sieved using individual plastic sterile sieves with 1 mm mesh size.

Quantification of heavy metals and metalloids (e.g., Fe, Cd, Cr, Cu, Mn, Hg, Ni, Zn, As) was carried out by ALS global (Loughrea, Ireland), using an aqua regia digest (EPA 3050b). To assess the total content of these determinants, samples were analysed using emission spectroscopy (ICP‐OES). For each sample, we quantified pH by suspending 1 g of soil in 5 mL of 0.01 m CaCl_2_ (Hendershot & Lalande 2008), which was shaken for 30 min and left to stand for 1 h, after which pH was measured using a Jenway 3510 pH meter (Stone, UK).

#### Siderophore production

The relationship between siderophore production, soil acidity and metal contamination was tested by screening a subset of clones for siderophore production. Siderophore production was necessarily measured under common garden conditions to avoid confounding effects of environmental variation if conducted *in situ*, causing both differential siderophore induction and metal‐chelating activities of the different soils themselves, which could directly affect the siderophore assay. For each sample, 1 g of soil was transferred to 6 mL of M9 solution in 30 mL glass vials, which were shaken for 2 h at 28 °C and 180 rpm, after which supernatant was plated onto LB agar. Thirty colonies per sample were randomly selected and grown for 48 h independently in 200 μL KB broth at 28 °C. A 2 μL sample from each colony was then spotted on blue‐tinted iron‐limited CAS agar plates (Schwyn & Neilands 1987) using a pin replicator. Plates were incubated at 28 °C for 48 h, after which we scored the presence of orange halos, a qualitative indicator of siderophore secretion, to obtain an estimate of the proportion of siderophore‐producing clones in each community.

#### DNA extractions and real time PCR

To determine how community abundance and composition varied across soils we extracted genomic DNA from 250 mg soil per sample, using MoBio Powerlyzer PowerSoil© DNA isolation kits (Carlsbad, CA, USA), following the manufacturer's protocol with the bead beating parameter set to 4500 rpm for 45 s. The integrity of DNA was confirmed using 1% TAE agarose gels stained with 1x Redsafe DNA Stain (20 000X); 5 samples were subsequently discarded, yielding 89 DNA samples in total.

Community density was quantified using real‐time PCR (StepOnePlus Real‐Time PCR, Applied Biosystems, Foster City, CA, USA) on 1 : 10 and 1 : 100 diluted samples with primers 16S rRNA 338F (ACT CCT ACG GGA GGC AGC AG) and 518R (ATT ACC GCG GCT GCT GG) (Øvreås & Torsvik 1998). Triplicates of each sample were run along gDNA standards (5 × 10^2−6^ 16S rRNA genes of *Pseudomonas fluorescens*) and non‐template controls. All assays were based on 15 μL reactions, using 1x Brilliant III Ultra‐Fast SYBR^®^ Green QPCR Master Mix (Agilent technologies, Santa Clara, CA, USA), 150 nm 338F and 300 nm 518R primers, 300 nm ROX and 100 ng μL^−1^ BSA. Thermal conditions were set to 3 min at 95 °C for initial denaturation, followed by 40 cycles of 5 s at 95 °C and 10 s at 60 °C (collection of fluorescent data), followed by a melting curve at 95 °C for 15 s, 60 °C for 1 min ramping up to 95 °C in steps of +0.3 °C for 15 s. Melting curves and confirmation of non‐template controls was analysed using StepOne Software (Applied Biosystems). Baseline corrections, Cq values and efficiencies (1.89 ± 0.07 and 1.89 ± 0.08 for standards and samples) were determined using LinRegPCR (Ruijter *et al*. 2009). 16S rRNA gene quantities were calculated using the one point calibration method (Brankatschk *et al*. 2012), corrected for variation in soil dry weight. Bacterial cell counts were estimated using ‘CopyRighter’ (Angly *et al*. 2014), which corrects for variation in lineage‐specific 16S gene copy numbers across samples. Note that this method does not account for unassigned OTUs.

#### Statistical analyses

Because of strong collinearity among heavy metals, we carried out a principal component analysis (PCA) on centred and scaled data. Most metals loaded positively on the first principal component (PC1; Fig. 2a), which was subsequently used as proxy for total metal contamination. To test how PC1 and pH affect the proportion of siderophore producers we used individual generalised linear models (GLMs) with a quasi‐binomial error structure. The effect of these environmental variables on bacterial densities was tested using individual GLMs on log_10_‐transformed data.

#### Sequencing, OTU picking and diversity analyses

Library preparation and sequencing was performed by the Centre for Genomic Research (University of Liverpool, Supplementary Methods).

Base‐calling and de‐multiplexing of indexed reads was performed using CASAVA (Illumina, San Diego, CA, USA) to produce 89 samples from the 1st lane of sequence data. Data were trimmed to remove (1) Illumina adapter sequences using Cutadapt (Martin 2011) and (2) low quality bases using Sickle (minimum quality score > 20), and (3) final reads < 10 bp. If both reads from a pair passed this filter, each was included in the R1 (forward reads) or R2 (reverse reads) file. If only one of a read pair passed this filter, it was included in the R0 (unpaired reads) file.

Sequences were processed using default parameters of the SmileTrain pipeline (https://github.com/almlab/SmileTrain/wiki/), including reads quality and chimera filtering, paired‐end joining, de‐replication and *de novo* distribution‐based clustering using USEARCH (Edgar 2010; http://www.drive5.com/usearch), Mothur (Schloss *et al*. 2009), Biopython, dbOTUcaller algorithms (Preheim *et al*. 2013; https://github.com/spacocha/dbOTUcaller) and custom scripts. We generated an OTU table that was filtered to minimise false OTUs using QIIME (Caporaso *et al*. 2010; http://qiime.org/) by removing OTUs observed < 10. We assigned taxonomy, post‐clustering, using the 97% reference OTU collection of the GreenGenes database (http://greengenes.lbl.gov). Taxonomy information was added to the OTU table using biom add‐metadata scripts (http://biom-format.org/). A total of 8 604 074 sequences were obtained, ranging from 39 253 to 192 455 reads per sample, with a median of 91 646. This dataset was clustered into 45 891 OTUs.

Diversity calculations were based on non‐rarefied OTU tables. β‐diversity was calculated using the Jensen–Shannon divergence metric (Fuglede & Topsoe 2004; Preheim *et al*. 2013), which is robust to sequencing depth variation. The R ‘phyloseq’ package (McMurdie & Holmes 2013) was used to transform the OTU table into relative abundances, which were square‐root‐transformed into Euclidean metrics (Legendre & Gallagher 2001). Finally, we used non‐metric multidimensional scaling (NMDS) plots (Shepard 1962; Kruskal 1964) to order bacterial community composition. Differences in community structure were tested using PERMANOVA (Anderson 2001), implemented using *adonis*() from the R ‘vegan’ package with 999 permutations.

To confirm that pH and PC1 shape community structure, we used K‐means partitioning algorithms (MacQueen 1967) implemented with *cascadaKM*() from the ‘vegan’ package with 999 permutations. K‐means is a completely independent way of binning samples. We Hellinger‐transformed (Rao 1995) the OTUs table using *decostand*(x. method=‘hellinger’) and tested whether our samples naturally clustered into 2–10 groups based on their composition using the Calinski–Harabasz index (Caliński & Harabasz 1974).

To investigate how environmental variables contributed towards explaining variation in community composition, we used multivariate regression tree analyses (MRT; Breiman *et al*. 1984; De'Ath 2002) for pH and PC1 separately, using the R ‘mvpart’ package (De'Ath 2007; Therneau *et al*. 2015). The OTU table was first Hellinger‐transformed (Rao 1995) before carrying out the analyses (Ouellette *et al*. 2012). After 200 cross‐validations (Breiman *et al*. 1984), we plotted and pruned the tree using the 1‐SE rule (Legendre & Legendre 2012) to select the least complex model. We used *rpart.pca*() from the ‘mvpart’ package to plot a PCA of the MRT.

α‐diversity was estimated using Shannon (Oksanen *et al*. 2010; ‘vegan’ package) and Chao1 (Vavrek & Larsson 2010; ‘fossil’ package) indices. We used *resample_estimate*() from the R ‘breakaway’ package (Willis & Bungle 2014) to account for sample size variability, setting the number of bootstraps to 500 with replacement. The relationship between α‐diversity and environmental variables was tested using *betta*() from the ‘breakaway’ package, which accounts for statistical errors associated with estimating α‐diversity indices.

### Copper‐addition experiment

#### Experimental design

To infer a causal relationship between toxic metals and siderophore production, we set up experimental compost communities. We isolated the community from fresh compost (Verve John Innes No. 1) by adding 40 g to 200 mL of M9 solution and incubating at 150 rpm at 28 °C for 24 h. Two ml (~ 5 × 10^7^ CFUs) of supernatant was subsequently used to seed twelve microbial communities in 90 mm Petri dishes containing 30 g of twice‐autoclaved compost. Hence, all treatments started off with the same community and level of siderophore production.

Microcosms were incubated at 26 °C and 75% humidity for 24 h, after which we supplemented six microcosms with 2 mL of filter‐sterilised 0.25 m CuSO_4_ or ddH_2_0. This concentration of CuSO_4_ hindered bacterial growth. Microcosms were incubated for 6 weeks. After 3 weeks, another 2 mL dose of CuSO_4_ or ddH_2_O was added where appropriate. Samples of the community were taken prior to copper amendment and 3–6 weeks post‐inoculation by transferring 1 g of compost to 6 mL of M9 solution in 30 mL glass vials. Vials were shaken for 2 h at 28 °C at 180 rpm, after which supernatants were frozen at − 80 °C in 25% glycerol.

#### Siderophore and copper resistance assays

To quantify siderophore production, 24 individual clones per treatment‐time combination were isolated by incubating supernatant on LB plates at 28 °C for 48 h. Individual colonies were then transferred to 2 mL of KB broth and grown for 48 h at 28 °C, after which the supernatant was assayed for the extent of iron chelation. Siderophore production was quantified using the liquid CAS assay described by Schwyn & Neilands (1987), with the modification that one volume of ddH_2_0 was added to the assay solution (Harrison & Buckling 2005). We used the following quantitative measure to obtain an estimate of siderophore production per clone: [1 − (A_*i*_/A_*ref*_)]/OD_*i*_, where OD_*i*_ = optical density at 600 nm and A_*i*_ = absorbance at 630 nm of the assay mixture *i* or reference mixture (KB + CAS; A_*ref*_). Note that CAS assays performed in iron‐limited KB (supplemented with 20 mm NaHCO_3_ and 100 μg mL^−1^ human apotransferrin) provided qualitatively similar results (data not shown).

All final time‐point clones were grown at 28 °C for 24 h, after which ~ 10^4^ CFUs were inoculated into 96‐well plate wells containing 200 μL of KB broth supplemented with or without a toxic dose of CuSO_4_ (6.17 mm). Clones were incubated statically at 28 °C for 48 h, and their OD was measured at 600 nm every 8–12 h to quantify growth (Varioskan Flash plate reader, Thermo Scientific, Waltham, MA, USA).

#### Sanger sequencing of 16S rRNA

The 16S rRNA gene of all assayed final‐time point clones was sequenced to confirm genus‐level identity: PCRs were performed in 25 μL reactions containing 1x DreamTaq Green PCR Master Mix (2X) (Thermo Scientific), 200 nm of the 27F and 1492R primers and 3 μL of 1 : 100 diluted culture that had undergone three freeze‐thaw cycles. The thermal cycling parameters were set to 94 °C for 4 min, followed by 35 cycles of 1 min at 94 °C, 30 s at 48 °C and 2 min at 72 °C, and a final extension of 8 min at 72 °C. Following Exo‐AP clean‐up, high quality samples were Sanger sequenced using the 27F primer (Core Genomic Facility, University of Sheffield).

The quality of all sequences was assessed using *plotQualityProfile*() from the R ‘dada2’ package (Callahan *et al*. 2016). Based on the obtained plots, sequences were trimmed in Genious to achieve an overall quality score > 35. Using Mother, sequences longer than 300 bp were aligned to the Silva.Bacteria.Fasta database, and taxonomy was classified using the RDP trainset 14 032015 as reference database.

#### Statistical analyses

The interactive effect of copper and time on mean siderophore production was tested using a linear mixed effects model (LME; ‘lme4’ R package; Bates *et al*. 2014) with copper × time (3–6 weeks post‐inoculation) as fixed categorical effects and random intercepts fitted for each community (*n *=* *12), and individual clones nested within communities (*n *=* *24), to account for temporal dependencies.

We used NMDS ordination plots to depict pair‐wise Bray–Curtis dissimilarities in genus‐level composition between microcosms. To test whether treatments differed significantly in their composition we used PERMANOVA with 999 permutations, and tested for equality of between‐treatment variance using permutation tests for homogeneity of multivariate dispersion.

To test for the effect of copper on metal tolerance, we used LME with *ln*(OD_Cu_/OD_KB_) as response variable, copper background as fixed effect and a random slope fitted for mean‐centred hours: random = ~ (Hours)|Community/Clone. The model thus accounts for intrinsic differences between communities, and nested clones, in their ability to tolerate toxic copper over time, and explicitly tests whether pre‐adaptation to copper increases mean copper tolerance. To test whether tolerance was directly mediated by variation in siderophore production, we replaced ‘copper background’ with clone‐specific siderophore production.

In general, full models were simplified by sequentially eliminating non‐significant terms (*P *>* *0.05), after which the significance of the explanatory variables was established using likelihood ratio tests. In case of significant differences, Tukey contrasts were computed using the ‘multcomp’ package (Hothorn *et al*. 2008), with α < 0.05. We used *R* Version 3.1.3 for all analyses (R Development Core Team; http://www.r-project.org).

## Results

### Foreign siderophores restore non‐producers fitness in toxic copper broth

**Figure 1:**
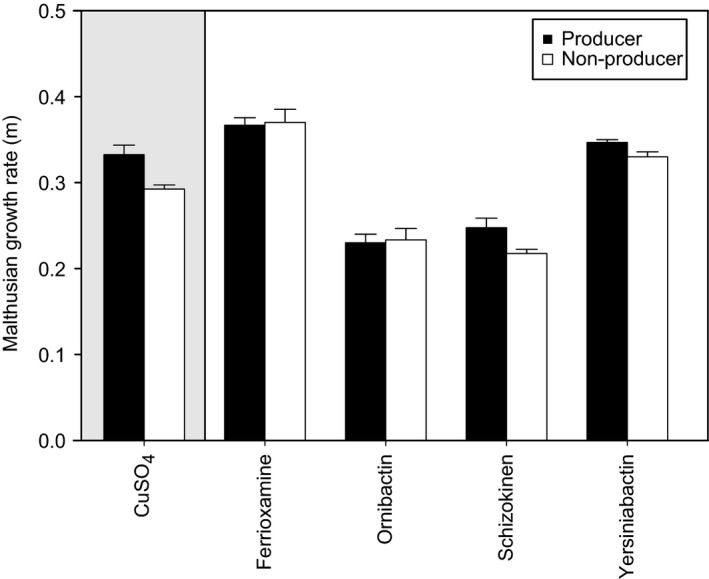
Siderophores act as an interspecific public good in toxic copper broth. Mean Malthusian growth rate (m) ± SE of a siderophore‐producing (black bars) and non‐producing (white bars) strain of *Pseudomonas aeruginosa* in toxic copper broth (0.6 mm CuSO_4_). The addition of heterospecific siderophores (ferrioxamine E, ornibactin, schizokinen and yersiniabactin) reduces mean growth differences between producing and non‐producing strains.

Non‐producer growth was significantly lower in toxic copper compared to that of the producing wild‐type strain of *P. aeruginosa* (*F*_1,6_ = 10.97, *P *=* *0.02; Fig. 1). Crucially, the addition of heterospecific siderophores significantly reduced mean growth differences between strains (one‐tailed t‐test: *t *=* *3.67, d.f. = 3, *P *=* *0.035; Fig. 1).

### Microbial diversity, abundance and siderophore production along a natural metal gradient

**Figure 2:**
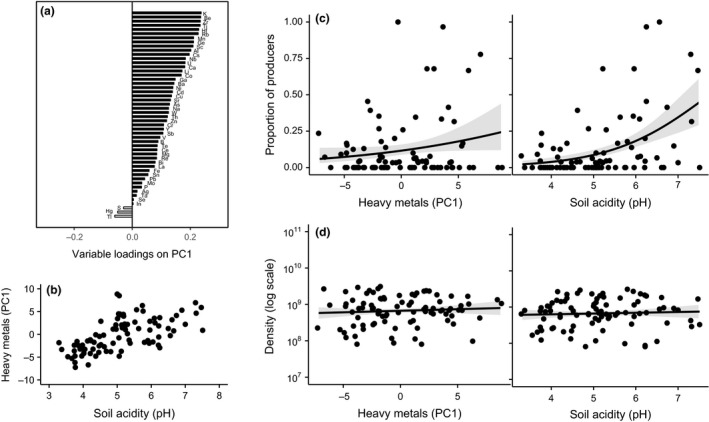
The effect of soil acidity and heavy metal contamination on microbial abundance and siderophore production in natural soils. (a) Heavy metal loadings on the first principal component (PC1), which explained 27% of the observed environmental variation; (b) Positive correlation between soil acidity (pH) and heavy metal contamination (PC1); (c) Proportion of siderophore producers and (d) microbial density (log_10_‐transformed bacterial cells g^−1^ soil) as a function of PC1 and pH. Lines and shaded area depict the fitted relationships ± SE.

**Figure 3:**
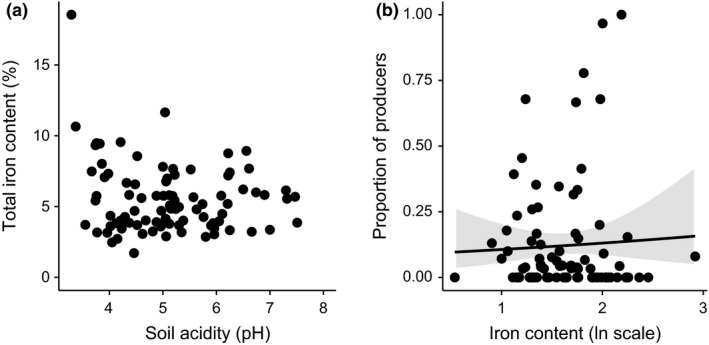
Relationship between soil acidity, iron and siderophore production. (a) Soil acidity (pH) and total iron content (%) do not co‐vary and (b) variation in total iron content (%, ln scale) does not affect the proportion of siderophore producers along a natural heavy metal gradient associated with historical mining activity. Line and shaded area depict the fitted relationship ± SE.

**Figure 4:**
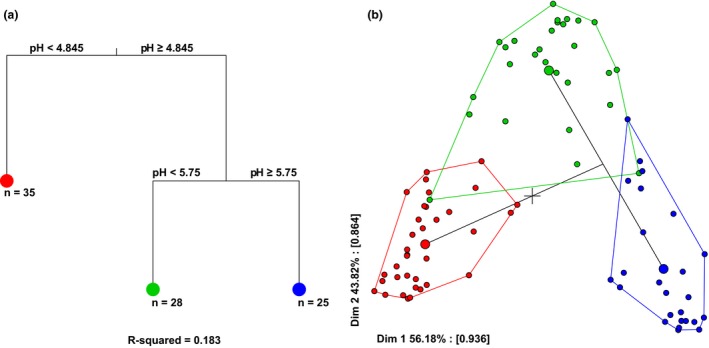
Community composition variation changes as a function of soil acidity. Multivariate regression tree analysis was used to estimate the impact of soil acidity (pH) and heavy metals (PC1) on community structure, indicating that pH is the main environmental driver explaining variation in community structure. The most parsimonious tree (a) shows that the community could be divided into three different leaves (coloured symbols) based on microbial abundance and composition. The composition within leaves is represented in a PCA plot (b), where small points represent individual samples and large points represent the group mean (within leaf). The most important taxa in each leaf are summarised in Supplementary Table S1.

We found that the proportion of siderophore‐producing isolates was significantly greater in more contaminated soils (PC1: χ^2^ = 4.42; d.f. = 1, *P *=* *0.04 Fig. 2c). Because contamination co‐varied with soil acidity (Pearson's correlation: *r *=* *0.61, d.f. = 86 and *P *<* *0.001; Fig. 2b), siderophore production also increased as a function of pH (χ^2^ = 28.16; d.f. = 1, *P *<* *0.001; Fig. 2c). Neither pH nor PC1 significantly affected microbial abundance (*F*_1,87/86_ = 0.01, *P *=* *0.99 for PC1 and pH; Fig. 2d). Note that total iron content neither co‐varied with pH (Pearson's correlation: *r *=* *0.03, d.f. = 86 and *P *=* *0.09; Fig. 3a) nor affected the proportion of siderophore producers (Fe: χ^2^ = 0.45; d.f. = 1, *P *=* *0.50; Fig. 3b). Both pH and PC1 predicted community structure: samples with similar range values of pH (PERMANOVA: R^2^ = 0.087, *P *<* *0.001) or PC1 (R^2^ = 0.065, *P *<* *0.001) had similar community composition. Because the explanatory power of these variables was relatively low (Fig. S1 in Supplementary Information), we performed a K‐means analysis, which showed that samples were naturally divided into 2–3 groups differing significantly in their PC1 or pH, respectively (Fig. S2). We used MRT to confirm these findings and observed that R^2^ was highest when pH was used as explanatory variable (pH: R^2^ = 0.183 and PC1: R^2^ = 0.085; Fig. 4). Alpha diversity was largely independent of PC1, but varied as a function of pH (Fig. S3; *P *<* *0.001 for both indices).

### The effect of copper on siderophore production in experimental communities

**Figure 5:**
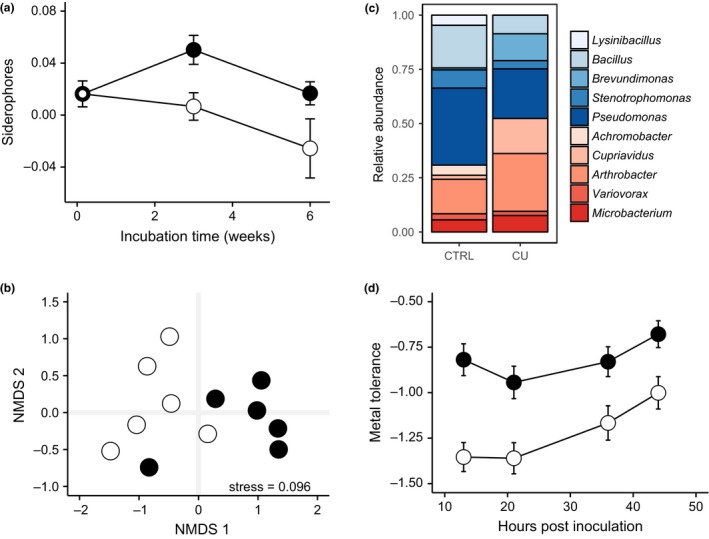
The effect of copper contamination on experimental microbial communities in compost. (a) Copper addition results in a net increase in mean per capita siderophore production ± SE over time, where open circles and black circles represent non‐contaminated and copper‐contaminated experimental communities, respectively; (b) non‐metric multidimensional scaling ordination plot depicting the pair‐wise Bray–Curtis dissimilarity between soil microcosms after 6 weeks of incubation (stress = 0.096). Points represent individual microcosms belonging to the non‐contaminated (open circles) and copper‐contaminated (black circles) treatment, such that microcosms similar in their genus‐level composition are ordinated closer together; (c) Relative abundance of the ten most common genera and their mean siderophore production. Genera are listed in order of their mean across‐treatment siderophore production, increasing from top to bottom, such that blue‐ and red genera are non‐producers and producers, respectively. See Supplementary Tables S2 for more details; (d) The effect of copper background (filled and open symbols are presence and absence of copper contamination, respectively) on metal tolerance, where more negative values indicate a stronger inhibitory effect of CuSO_4_ on bacterial growth. Bars denote 1 SE.

Our assay of siderophore production along a natural metal gradient showed that siderophore production was greater in more contaminated soils. However, it remains unclear whether metals are a significant driver explaining variation in siderophore production. Notably, pH is an important predictor of soil bacterial diversity and composition (e.g., Fierer & Jackson 2006; Griffiths *et al*. 2011), and correlated positively with contamination, making any interpretation ambiguous. To determine a causal link between metals and siderophore production, we carried out an experiment and characterised and measured siderophore production of multiple clones as well as their metal tolerance. We found that mean siderophore production was significantly greater in communities subjected to copper contamination (LME: copper effect: χ^2^ = 6.91; d.f. = 1; *P *<* *0.01; Fig. 5a). Note that overall siderophore production decreased through time (time effect: χ^2^ = 16.02; d.f. = 1; *P *<* *0.001; Fig. 5a), independent of treatment (time × treatment effect: χ^2^ = 0.001; d.f. = 1; *P *=* *0.98). Soil acidity marginally increased following copper contamination (mean pH ± SE after 3 and 6 weeks of incubation in control = 7.13 ± 0.05_,_ 7.09 ± 0.02 and in copper = 6.90 ± 0.04, 6.60 ± 0.05), indicating that siderophore production was greater in more acidic compost.

We identified clones at the genus‐level to explore the role of ecological sorting in driving siderophore production. Community composition varied significantly between treatments (PERMANOVA: *F*_1,11_ = 3.88, *P *=* *0.015; multivariate dispersion: *F*_1,11_ = 0.021, *P *=* *0.91; Fig. 5b), with siderophore‐producing genera being selectively favoured in copper‐contaminated compost (Fig. 5c and Supplementary Table S2). Crucially, clones isolated from copper‐contaminated communities were significantly less inhibited when grown in toxic copper broth compared to those from non‐contaminated communities (LME: χ^2^ = 6.80; d.f. = 1; *P *<* *0.01; Fig. 5d), which was mediated by increased siderophore production (LME: χ^2^ = 16.68; d.f. = 1; *P *<* *0.001).

## Discussion

In this study, we investigated how heavy metals affected ecological selection for siderophore production – an interspecific microbial public good – across a natural contamination gradient and during a controlled experiment in compost. We hypothesised there could be selection for both increased and decreased siderophore production, because of the detoxifying effect of siderophores and the potential for interspecific exploitation, respectively. Our findings suggest that the presence of toxic metals resulted in net ecological selection for taxa that produced large amounts of siderophore, although this does not rule out the possibility that some exploitation occurred. We also confirmed that bacteria producing more siderophores suffered less growth inhibition in toxic copper broth.

Ecological selection for increased siderophore production contrasts with previous *in vitro* within‐species (*P. aeruginosa*) results, in which non‐producing ‘cheats’ were able to outcompete siderophore producers in copper‐contaminated broth (O'Brien *et al*. 2014), resulting in a net reduction in siderophore production in the presence of toxic metals. A key reason for this difference is likely to be the spatial structure in soil/compost resulting in localised detoxification, such that producers and their immediate neighbours gain the most from siderophores (Hamilton 1964; West & Buckling 2003; Buckling *et al*. 2007; West *et al*. 2007; Lujan *et al*. 2015). Hence, low siderophore producers should experience more of the toxic metal effect. Limited dispersal would also lead to immediate neighbours having a higher probability of being conspecifics – a likely reason as to why taxa that typically produce more siderophores dominated metal‐contaminated communities. Direct comparison of intra‐ and inter‐specific changes in siderophore production in soil would tease apart the differing roles of spatial and community structure in determining these results.

Siderophore production decreased in all our experimental communities over time, which is likely caused by novel abiotic selection pressures resulting from the laboratory conditions. We also cannot rule out the possibility that non‐producers did in fact benefit from siderophores produced by other community members. However, as the decrease occurred in both copper and non‐copper environments, this reduction cannot be explained by exploitation of detoxifying siderophores. That is not to say that this exploitation does not play a role in the observed levels of siderophore production, but that the beneficial effects of siderophores to the producers outweigh these costs. This is analogous to the evolution of collective antibiotic resistance in microbial populations (Lee *et al*. 2010; Vega & Gore 2014), where resistant cells enhance the survival capacity of the overall population by allowing ‘weaker’ cells to endure more antibiotic stress than they could in isolation.

In our survey of a former mining area, soil acidity and total contamination positively co‐varied, with both prolonged metal leaching in acidic soils and precipitation in more basic soils likely contributing to this pattern (Alloway 1990; Adriano 2001). This covariance may well have contributed to the observed patterns. First, acidity is a major determinant of microbial community composition (e.g., Fierer & Jackson 2006; Griffiths *et al*. 2011), hence pH‐mediated selection may have indirectly favoured taxa that produce siderophores in larger amounts. Second, acidity affects metal speciation and bio‐availability to microbes in variable ways (Lofts *et al*. 2004; Gobran & Huang 2011), with iron becoming largely insoluble at pH > 6.5 (Guerinot 1994). As such, increased siderophore production in basic soils, which also had the highest metal concentrations, may have been driven by selection imposed by iron limitation. However, our experimental manipulations, where the same compost community was propagated with and without copper, strongly suggest a direct effect of metal‐imposed selection on siderophore production. This manipulation did have a small effect on pH (copper decreased pH from *c*. 7.1 to 6.6), but in this case there was negative, rather than positive, covariance.

It was initially surprising to find that microbial densities were similar along the contamination gradient; several studies have demonstrated that toxic metals reduce microbial abundance (reviewed in Giller *et al*. 1998). These differences may perhaps reflect relatively low concentrations of biologically available metals in our study; we only measured total metal content. Moreover, given the mining history of our focal site, microbes are likely to be relatively well adapted to toxic metals: selection of taxa with increased copper tolerance occurred very rapidly in our experiment. Note that other more direct resistance mechanisms, in addition to siderophore production, such as metal reduction and reduced cell permeability (Nies 1999; Bruins *et al*. 2000; Valls & De Lorenzo 2002), were not investigated here and hence their importance relative to siderophores in determining metal resistance is unknown.

Human‐imposed metal contamination is a major problem for natural ecosystems. Several studies have noted that addition of siderophores or siderophore‐producing microbes could aid in detoxifying contaminated soils, particularly when combined with the use of hyper‐accumulating plants, which commonly extract metals more efficiently when bound to siderophores (Lebeau *et al*. 2008; Dimkpa *et al*. 2009). Crucially, hyper‐accumulating plants take up siderophore‐metal complexes before metals flow back in the system following siderophore decay. Our results provide some key insights into the optimal use of siderophores for phytoremediation. The addition of high siderophore‐producing bacteria following recent contamination events is likely to be effective, because these organisms should have a selective advantage and hence contribute to increasing community‐level siderophore production. However, siderophore addition is unlikely to significantly improve phytoremediation of historically contaminated sites, in which siderophore production will already have been stabilised by selection. The direct addition of siderophores, while providing a short‐term benefit, may actually result in longer‐term negative effects on phytoremediation regardless of length of time since contamination, as selection for siderophore production is relaxed. More generally, our results highlight that interspecific public goods production can be maintained at high levels in natural microbial communities, despite the potential of exploitation by cheating non‐producers.

## Conflict of Interest

We declare no conflict of interest.

## Authorship

EH, SOB, AML, DJH, EMvV, AB conceived and designed the experiment. DJH provided new perspectives. EH, SOB, FB, AML collected the data. EH, FB, NT, DJH carried out the data analyses. EH & AB wrote the first draft of the manuscript, and all authors contributed substantially to revisions.

## Data Accessibility Statement

The research materials supporting this publication can be accessed at the Dryad Digital Repository (https://doi.org/10.5061/dryad.8c0t7). The raw sequence data have been deposited in the GenBank SRA database (BioProject accession no. PRJNA414950).
