# Supplementary material for: Ecological selection of siderophore‐producing microbial taxa in response to heavy metal contamination
Source: Ecol Lett. 2017 Nov 21;21(1):117–27. doi: 10.1111/ele.12878 (PMC5765521; doi:10.1111/ele.12878)
Supplement: Supplementary file 1 [file ELE-21-117-s001.docx]

**Supplementary information**

**Figure S1. Changes in community composition in relation to soil acidity and heavy metal contamination.** NMDS ordination plot depicting pairwise JSD distances along **(A)** soil acidity (pH) and **(B)** heavy metal (PC1) gradients.

**Figure S2.** **K-means partitioning.** We used the K-means unsupervised learning algorithms to classify our dataset into clusters based on the OTU table. We tested 2-10 clusters and repeated the analysis 999 times. K-means cascade plot **(A)** depicting the group attributed for each sample (object). The Calinski-Harabasz index was used to determine the optimal configuration of clusters. **(B)** representing how samples belonging to the K-means groups are distributed according to soil acidity (pH) and heavy metal contamination (PC1). Comparisons were performed using a Mann-Whitney test (*P < 0.05. **P < 0.01. ***P < 0.001).

**Figure S3. Alpha diversity across a soil acidityand metal contamination gradient.** Two alpha diversity metrics were employed: the Shannon index (A-B) and the Chao1 richness (C-D) to compare the alpha diversity along a soil acidity (pH, A-C) and heavy metal (PC1, B-D) gradient.

**Table 1.** Siderophore production (i.e. ability to chelate iron in KB broth) after six weeks of incubation in compost microcosms. The table summarizes mean and variation in siderophore production for ten genera commonly present in non-contaminated and copper-contaminated compost.

**Figure S1**


**Figure S2.**

**Figure S3**
