# Supplementary material for: Ecological selection of siderophore‐producing microbial taxa in response to heavy metal contamination
Source: Ecol Lett. 2017 Nov 21;21(1):117–27. doi: 10.1111/ele.12878 (PMC5765521; doi:10.1111/ele.12878)
Supplement: Supplementary file 2 [file ELE-21-117-s002.docx]

**Supplementary Tables**

**Table S1.** Multivariate regression tree (MRT) analysis was used to estimate the impact of pH on microbial community structure.

**Table S2.** Per capita siderophore in ten genera commonly found in experimental communities following 6 weeks of incubation.

**Table S1.** Multivariate regression tree (MRT) analysis was used to estimate the impact of pH on microbial community structure. The most parsimonious tree shows that the community could be divided into three different leaves (red, green and blue) and the most important taxa in each leaf are summarized below (see Fig. 3 for more detailed description).

| **Scores** | **OTU ID** | **Leaf** | **Taxa** |
| --- | --- | --- | --- |
| 0.747 | seq7251 | Red | k_Bacteria\|p_Planctomycetes\|c_Phycisphaerae\|o_WD2101\|f_Unclassified\|g_Unclassified\|s_Unclassified |
| 0.660 | seq5199 | Red | k_Bacteria\|p_WPS-2\|c_Unclassified\|o_Unclassified\|f_Unclassified\|g_Unclassified\|s_Unclassified |
| 0.638 | seq7379 | Red | k_Bacteria\|p_AD3\|c_JG37-AG-4\|o_Unclassified\|f_Unclassified\|g_Unclassified\|s_Unclassified |
| 0.636 | seq1593 | Red | k_Bacteria\|p_Planctomycetes\|c_Planctomycetia\|o_Gemmatales\|f_Isosphaeraceae\|g_Unclassified\|s_Unclassified |
| 0.634 | seq5398 | Red | k_Bacteria\|p_AD3\|c_JG37-AG-4\|o_Unclassified\|f_Unclassified\|g_Unclassified\|s_Unclassified |
| 0.626 | seq5164 | Red | k_Bacteria\|p_AD3\|c_JG37-AG-4\|o_Unclassified\|f_Unclassified\|g_Unclassified\|s_Unclassified |
| 0.603 | seq6200 | Red | k_Bacteria\|p_Acidobacteria\|c_Acidobacteriia\|o_Acidobacteriales\|f_Koribacteraceae\|g_Unclassified\|s_Unclassified |
| 0.593 | seq1629 | Red | k_Bacteria\|p_Actinobacteria\|c_Actinobacteria\|o_Actinomycetales\|f_Unclassified\|g_Unclassified\|s_Unclassified |
| 0.591 | seq5765 | Red | k_Bacteria\|p_Actinobacteria\|c_Acidimicrobiia\|o_Acidimicrobiales\|f_Unclassified\|g_Unclassified\|s_Unclassified |
| 0.578 | seq5457 | Red | k_Bacteria\|p_Acidobacteria\|c_Acidobacteriia\|o_Acidobacteriales\|f_Acidobacteriaceae\|g_Unclassified\|s_Unclassified |
| 0.664 | seq20403 | Green | k_Bacteria\|p_Proteobacteria\|c_Alphaproteobacteria\|o_Rhizobiales\|f_Unclassified\|g_Unclassified\|s_Unclassified |
| 0.657 | seq19973 | Green | k_Bacteria\|p_Proteobacteria\|c_Alphaproteobacteria\|o_Rhizobiales\|f_Unclassified\|g_Unclassified\|s_Unclassified |
| 0.653 | seq3572 | Green | k_Bacteria\|p_Acidobacteria\|c_Acidobacteria-6\|o_iii1-15\|f_Unclassified\|g_Unclassified\|s_Unclassified |
| 0.649 | seq3027 | Green | k_Bacteria\|p_Chloroflexi\|c_Ellin6529\|o_Unclassified\|f_Unclassified\|g_Unclassified\|s_Unclassified |
| 0.638 | seq30456 | Green | k_Bacteria\|p_Proteobacteria\|c_Betaproteobacteria\|o_MND1\|f_Unclassified\|g_Unclassified\|s_Unclassified |
| 0.636 | seq3171 | Green | k_Bacteria\|p_Actinobacteria\|c_Thermoleophilia\|o_Gaiellales\|f_Gaiellaceae\|g_Unclassified\|s_Unclassified |
| 0.634 | seq30539 | Green | k_Bacteria\|p_Acidobacteria\|c_Acidobacteria-6\|o_iii1-15\|f_Unclassified\|g_Unclassified\|s_Unclassified |
| 0.620 | seq20133 | Green | k_Bacteria\|p_Acidobacteria\|c_Acidobacteria-6\|o_iii1-15\|f_mb2424\|g_Unclassified\|s_Unclassified |
| 0.614 | seq2733 | Green | k_Bacteria\|p_Acidobacteria\|c_Acidobacteria-6\|o_iii1-15\|f_Unclassified\|g_Unclassified\|s_Unclassified |
| 0.686 | seq985 | Blue | k_Bacteria\|p_Chloroflexi\|c_Thermomicrobia\|o_JG30-KF-CM45\|f_Unclassified\|g_Unclassified\|s_Unclassified |
| 0.669 | seq176 | Blue | k_Bacteria\|p_Chloroflexi\|c_Ktedonobacteria\|o_Thermogemmatisporales\|f_Thermogemmatisporaceae\|g_Unclassified\|s_Unclassified |
| 0.661 | seq39 | Blue | k_Bacteria\|p_Chloroflexi\|c_Ellin6529\|o_Unclassified\|f_Unclassified\|g_Unclassified\|s_Unclassified |
| 0.660 | seq97 | Blue | k_Bacteria\|p_Chloroflexi\|c_Ktedonobacteria\|o_Thermogemmatisporales\|f_Thermogemmatisporaceae\|g_Unclassified\|s_Unclassified |
| 0.646 | seq169 | Blue | UnassignedUnclassified |
| 0.644 | seq319 | Blue | k_Bacteria\|p_Chloroflexi\|c_Ktedonobacteria\|o_Thermogemmatisporales\|f_Thermogemmatisporaceae\|g_Unclassified\|s_Unclassified |
| 0.636 | seq14280 | Blue | k_Bacteria\|p_Chloroflexi\|c_Ktedonobacteria\|o_Thermogemmatisporales\|f_Thermogemmatisporaceae\|g_Unclassified\|s_Unclassified |
| 0.626 | seq14114 | Blue | k_Bacteria\|p_Planctomycetes\|c_Planctomycetia\|o_Gemmatales\|f_Gemmataceae\|g_Unclassified\|s_Unclassified |
| 0.620 | seq15 | Blue | k_Bacteria\|p_Verrucomicrobia\|c_[Spartobacteria]\|o_[Chthoniobacterales]\|f_[Chthoniobacteraceae]\|g_DA101\|s_Unclassified |
| 0.616 | seq508 | Blue | k_Bacteria\|p_Chloroflexi\|c_Thermomicrobia\|o_Ellin6537\|f_Unclassified\|g_Unclassified\|s_Unclassified |

**Table S2. Per capita siderophore production (mean, variation and number of clones assayed)^¶^ in ten commonly found genera after six weeks of incubation in copper-contaminated and non-contaminated compost.**

| **Genus** | **Copper-contaminated** | | | **Non-contaminated** | | | **Overall mean^§^** |
| --- | --- | --- | --- | --- | --- | --- | --- |
|  | *Mean* | *Variation* | *n* | *Mean* | *Variation* | *n* |  |
| *Lysinibacillus* | NA | NA | NA | -0.083 | 0.009 | *5* | **-0.083** |
| *Bacillus* | 0.003 | 0.020 | *8* | -0.160 | 0.057 | *21* | **-0.079** |
| *Brevundimonas* | -0.032 | 0.012 | *14* | -0.041 | NA | *1* | **-0.037** |
| *Stenotrophomonas* | -0.023 | 0.001 | *4* | -0.028 | 0.004 | *9* | **-0.025** |
| *Pseudomonas* | 0.039 | 0.003 | *24* | -0.051 | 0.015 | *38* | **-0.006** |
| *Achromobacter* | NA | NA | NA | 0.016 | 0.001 | *5* | **0.016** |
| *Cupriavidus* | 0.010 | 0.001 | *17* | 0.032 | 0.001 | *2* | **0.021** |
| *Arthrobacter* | 0.026 | 0.008 | *28* | 0.028 | 0.006 | *17* | **0.027** |
| *Variovorax* | 0.101 | 0.001 | *2* | 0.048 | 0.011 | *4* | **0.075** |
| *Microbacterium* | 0.057 | 0.009 | *8* | 0.147 | 0.021 | *6* | **0.102** |

**^¶^** Siderophore production was measured as the ability of individual clones to chelate iron in KB broth.

§ The overall mean is depicted in Fig. 5C of the manuscript.
