# Supplementary material for: Ecological selection of siderophore‐producing microbial taxa in response to heavy metal contamination
Source: Ecol Lett. 2017 Nov 21;21(1):117–27. doi: 10.1111/ele.12878 (PMC5765521; doi:10.1111/ele.12878)
Supplement: Supplementary file 3 [file ELE-21-117-s003.docx]

**Supplementary Methods**

The Centre for Genomic Research (University of Liverpool) used the primers described by Caporaso et al (2011) to amplify the V4 region of 16S ribosomal RNA. 5 μl of DNA entered a first round of PCR with cycle conditions 20s at 95^o^C, 15s at 65^o^C, 30s at 70^o^C for 10 cycles, followed by a final 5min extension at 72^o^C. The sequence of the primers is:

F:5'ACACTCTTTCCCTACACGACGCTCTTCCGATCTNNNNNGTGCCAGCMGCCGCGGTAA3'

R: 5'GTGACTGGAGTTCAGACGTGTGCTCTTCCGATCTGGACTACHVGGGTWTCTAAT3'

The primer design incorporates a recognition sequence to allow a secondary nested PCR step. Samples were first purified with Axygen SPRI Beads before entering the second PCR performed to incorporate Illumina sequencing adapter sequences containing indexes (i5 and i7) for sample identification. 15 cycles of PCR were performed using the same conditions as above for a total of 25 cycles. Samples were purified using Axygen SPRI Beads before being quantified using Qubit and assessed using the Fragment Analyzer. Successfully generated amplicon libraries were taken forward. These final libraries were pooled in equimolar amounts using the Qubit and Fragment Analyzer data and size selected on the Pippin prep using a size range of 300-600 bp. The quantity and quality of each pool was assessed by Bioanalyzer and subsequently by qPCR using the Illumina Library Quantification Kit from Kapa on a Roche Light Cycler LC480II according to manufacturer's instructions. The template DNA was denatured according to the protocol described in the Illumina cBot User guide and loaded at 8.5 pM concentration. To help balance the complexity of the amplicon library 15% PhiX was spiked in. The sequencing was carried out on one lane of an Illumina MiSeq at 2x250 bp paired-end sequencing with v2 chemistry. R^2^=0.21.
